# Loss of has-miR-337-3p expression is associated with lymph node metastasis of human gastric cancer

**DOI:** 10.1186/1756-9966-32-76

**Published:** 2013-10-16

**Authors:** Zishu Wang, Jin Wang, Yan Yang, Bo Hao, Rui Wang, Yumei Li, Qiong Wu

**Affiliations:** 1 Department of Medical Oncology, The First Affiliated Hospital of Bengbu Medical College, 287 Changhuai Road, Bengbu 233004, Anhui, China; 2 Department of Oncology, Changzheng Hospital, The Second Military Medical University, Shanghai 200433, China; 3 Department of Gastrointestinal Surgery, The First Affiliated Hospital of Bengbu Medical College, Bengbu, Anhui 233004, China

**Keywords:** Gastric cancer, miRNA, Metastasis, hsa-miR-337-3p, miRNA array

## Abstract

**Background:**

Metastasis is the major cause of cancer-related death in patients with gastric cancer, and aberrant expression of various microRNAs (miRNAs) is associated with cancer metastasis.

**Methods:**

Profiling of differentially expressed miRNAs was performed in three cases of primary gastric cancer and the corresponding metastatic lymph node tissues. Then, the five most altered miRNAs were further verified in 16 paired samples. Two of these five miRNAs were further assessed for their effects on the regulation of gastric cancer cell growth and invasion.

**Results:**

The miRNA profile data showed 151 upregulated miRNAs (≥ 1.5-fold) and 285 downregulated miRNAs (≤ 0.67-fold) in the metastatic tissues compared to the primary gastric cancer tissues. Among these five miRNAs (i.e., hsa-miR-508-5p, hsa-miR-30c, hsa-miR-337-3p, hsa-miR-483-5p, and hsa-miR-134), expression of hsa-miR-337-3p and hsa-miR-134 was significantly downregulated in these 16 lymph node metastatic tissues compared to their primary tumor tissues (P<0.05) and in nine gastric cancer cell lines compared to the nonmalignant GES cell line. Furthermore, induction of hsa-miR-134 or hsa-miR-337-3p expression did not dramatically affect gastric cancer cell proliferation, but transfection of the hsa-miR-337-3p mimic did reduce gastric cancer cell invasion capacity.

**Conclusions:**

These findings indicate that hsa-miR-337-3p plays a role in the reduction of gastric cancer cell invasion capacity, and further studies on the mechanism of hsa-miR-337-3p in gastric cancer metastasis are warranted.

## Background

Gastric cancer is a significant health problem, accounting for approximately one million new cases and more than 700,000 cancer-related deaths annually in the world [1-3]. Although the incidence of gastric cancer has substantially decreased in most parts of the world for the past few decades, partially due to consumption of more fresh fruits and reduction of *Helicobacter pylori* infection in the population [1-3], to date, a large number of patients with gastric cancer are still diagnosed at advanced stages, which makes curative surgery difficult. Approximately 80% of such patients will die within a short period of time due to regional recurrence or distant metastasis [4,5]. Tumor metastasis involves a complex series of steps in which tumor cells leave their original site and spread to distant organs or tissues. Metastasis is the major cause of cancer-related death, and the underlying molecular mechanisms are not fully understood. However, it is known that increased migration and invasion of primary cancer cells are the primary means by which gastric cancer cells spread to distant sites. Thus, there is an urgent need and a great clinical interest to better understand the molecular mechanisms responsible for gastric cancer metastasis in order to improve the outcome of gastric cancer patients.

To this end, our recent research on gastric cancer has focused on microRNAs (miRNAs), which are small, single-stranded noncoding RNA molecules of 19–23 nucleotides in length that are able to post-transcriptionally regulate target gene expression [6]. So far, several hundred miRNAs have been identified in plants, animals, and even viral RNA genomes. In humans, miRNAs regulate many cellular processes through binding to 3′-untranslated regions (UTRs) and other regions of protein-coding mRNA sequences of their target mRNAs to cause mRNA degradation or inhibit its translation [7]. Thus, altered miRNA expression plays a role in tumor development and progression, such as tumor cell proliferation, invasion, and metastasis [8]; in addition, certain miRNAs also can predict the prognosis of various cancers, including gastric, breast, lung, and prostate cancers [9,10]. In gastric cancer, aberrant expression of miRNAs has been linked to tumor metastasis; for example, plasma levels of miR-223, miR-21, miR-218, and miR-25 have been linked to gastric cancer metastasis [11,12]. Furthermore, elevated miR-21 expression is associated with lymph node metastasis of gastric cancer [13]. Thus, these miRNAs could be useful as biomarkers to predict gastric cancer lymph node metastasis. In addition, miR-625 expression is significantly downregulated and inversely associated with lymph node metastasis of gastric cancer [14]. Therefore, in the present study, we first performed miRNA array analysis to profile differentially expressed miRNAs between primary and secondary gastric cancer tissues. We found that the expression of hsa-miR-134 and hsa-miR-337-3p was significantly less in metastatic lymph node tissues than in primary tumors of gastric cancer. Next, we investigated the effects of hsa-miR-134 or hsa-miR-337-3p on the inhibition of gastric cancer cell growth and invasion. The results of this study may be useful to find potential therapeutic agents to inhibit gastric cancer metastasis.

## Methods

### Tissue samples

In this study, samples of human primary gastric cancer and the corresponding metastatic lymph node tissues were collected from 19 patients and stored in liquid nitrogen until use. The demographic data of these patients are shown in Table 1. The institutional review board of the First Affiliated Hospital of Bengbu Medical College approved our protocol, and the patients signed a consent form to participate in this study.

**Table 1:** List of miRNAs found to be less expressed in metastatic lymph node tissues compared to the corresponding primary gastric cancer tissues as determined by miRNA microarray analysis

**miRNA**	**Relative expression level (ΔΔCT, Mean ± SD)**		
	**GC**	**LN**	***P *value by paired T-test**
hsa-miR-483-5p	−1.08±3.08	−2.49±3.56	0.241
hsa-miR-508-5p	−5.49±1.64	−7.48±1.96	0.069
hsa-miR-30c	4.37±3.70	2.27±5.47	0.058
hsa-miR-134	0.92±4.48	−1.50±4.19	0.022*
hsa-miR-337-3p	−3.67±3.32	−6.04±2.73	0.005*

Gastric cancer (GC) vs. lymph nodes (LN); n=3; *P<0.05.

### RNA isolation and miRNA microarray profiling

Total cellular RNA was isolated from tissue specimens using TRIzol® reagent (Invitrogen, Carlsbad, CA). Briefly, the frozen tissues were homogenized by using a biopulverizer with Mini-Bead-Beater-16 and added to TRIzol® reagent for RNA isolation according to the manufacturer’s instructions. The RNA purity was assessed by measuring the absorption rate at 260 nm and at 280 nm in a NanoDropND-1000 spectrophotometer (A260/A280 ratio of 1.8–2.1 was considered acceptable), and the RNA integrity number (RIN) was detected by an Agilent 2000 analyzer (RIN≥8.0).

Next, these RNA samples of human primary gastric cancer and the corresponding metastatic tissues were reversely transcribed into cDNA, labeled with Hy3 and Hy5, and used as probes for miRNA profiling using the miRCURYTM LNA system (MicroRNA array V10.0 whole list, LC Sciences, Houston, TX). After bioinformatics analysis of the primary gastric cancer and metastatic tissue samples, the differentially expressed miRNAs were identified.

### Quantitative reverse transcription polymerase chain reaction (qRT-PCR)

To confirm some of these differentially expressed miRNAs, tumor tissues were harvested and stored in RNAlater solution (Ambion, Austin, TX). Total cellular RNA was isolated from RNAlater-fixed tumor tissues or fresh cultured cells by using the mirVana™ miRNA isolation kit (Ambion, Austin, TX) and reversely transcribed into cDNA with the TaqMan® MicroRNA reverse transcription kit (Applied Biosystems, Foster City, CA). Taqman gene expression assays (Applied Biosystems) were used to assess expression levels of hsa-miR-508-5p, hsa-miR-337-3p, hsa-miR-30c, hsa-miR-483-5p, hsa-miR-134, and U6 in tissues or cultured cells by the 7900HT fast real-time PCR system (Applied Biosystems, Darmstadt, Germany). Relative expression levels of each miRNA were calculated using the ΔΔCT method after normalization with U6 levels (an internal control).

### Cell lines and culture

A nonmalignant GES cell line and nine human gastric cancer cell lines (SNU1, SNU5, AGS, HGC-27, BGC-823, MGC-803, SGC-7901, MKN-28, and MKN-45) were originally purchased from the Cell Bank of the Chinese Academy of Science (Shanghai, China), stored, recovered, and used at an early passage from cryopreservation in liquid nitrogen. These cells were maintained in RPMI 1640 medium containing 10% fetal bovine serum (FBS), 2 mM L-glutamine, penicillin (100 units/mL), and streptomycin (100 μg/mL). All cell lines were cultured in 6-well plates in humidified air supplemented with 5% CO_2_ at 37°C. After cell culture for 48 h, total RNAs were isolated and used for qRT-PCR, respectively.

### Design of siRNA oligonucleotides and transfection into tumor cells

The oligonucleotides were synthesized by GenePharma (GenePharma, Shanghai, China) and hsa-miR-134 and hsa-miR-337-3p mimics and inhibitors were purchased from Ambion (Austin, TX). miRNA mimics and inhibitors, and siRNA transfection was performed using FuGene® HD transfection reagent (Roche, Mannheim, Germany). In brief, cells were plated in a 24-well plate and grown to 50% confluency. Then, 1 μl of FuGene® HD transfection reagent was diluted in 50 μl of Opti-MEM® I Reduced Serum Medium (GIBCO BRL). After that, 100 pmol of siRNA oligomer was diluted in 50 μl of Opti-MEM® I Reduced Serum Medium without serum (final concentration of oligonucleotides when added to the cells was 20 μM according to the protocol of the manufacture and the preliminary experiments). The FuGene® HD transfection complex and the diluted oligonucleotides were mixed gently and incubated at room temperature. After incubation for 20 min, the complexes were added to each well containing cells and medium. The cells were incubated for 6 h at 37°C in a CO_2_ incubator prior to testing for transfection.

### Cell proliferation assay

A CCK-8 (Dojindo, Shanghai, China) cell proliferation assay was used to assess cell proliferation, according to the manufacturer’s protocol. Briefly, cells were grown and transfected with hsa-miR-134 and hsa-miR-337-3p mimics and inhibitors (50 nM miRNA scrambled control or miRNA mimic or 200 nM miRNA inhibitor scrambled control or miRNA inhibitor) for 48 h [15], detached, and cultured in triplicate in 96-well cell culture plates. At the end of the experiments, the cells were washed with phosphate-buffered saline (PBS), fixed in 1% glutaraldehyde, and stained with 10% CCK-8. The optical density (OD) at 450 nm was directly measured with a Bio-Rad microplate reader (Hercules, CA).

### Tumor cell invasion assay

Gastric cancer cell invasion capacity was assessed by using a two-chamber migration system. The upper compartment was inserted into the lower compartment of the BD BioCoat control inserts (BD Discovery Labware, Bedford, MA), 5 × 10^4^ cells in 0.1 mL of serum-free medium containing 1% bovine serum albumin (BSA) were seeded into the upper compartment, and the lower compartment was filled with normal culture medium supplemented with 20% FBS. After incubation for 24 h, cells were wiped away from the upper surface and the cells on the lower surface, which represented the cells that migrated through the control insert membrane, were fixed and stained with crystal violet (Sigma). The number of cells that migrated completely across the filter was determined in five random fields (×400 magnification) for each experiment. Each condition was assayed in triplicate, and each experiment was repeated at least three times.

### Statistical analysis

All experiments were repeated at least three times on different occasions. The results are presented as the mean ± standard deviation (SD) for all values. A paired Student’s t-test was used to evaluate statistically significant differences between the parameters of the primary tumor tissues and the metastatic tumor tissues. Analysis of variance (ANOVA) was used for miRNA selection from the miRNA microarray study. P<0.05 was considered statistically significant.

## Results

### miRNA expression profiles of gastric cancer tissues and the corresponding metastatic lymph node tissues

In this study, we first profiled differentially expressed miRNAs between gastric cancer and the corresponding metastatic lymph node tissues. After profiling three cases of paired tissue samples (the pathology of these cancer tissues is listed in Additional file 1: Figure S1), we found 151 upregulated miRNAs (≥1.5-fold; Additional file 2: Table S1) and 285 downregulated miRNAs (≤0.67-fold) in the metastatic tissues compared to the primary gastric cancer tissues (Additional file 2: Table S1). Specifically, expression of hsa-miR-508-5p, hsa-miR-483-5p, hsa-miR-134, hsa-miR-30c, and hsa-miR-337-3p was reduced in all three metastatic cancer tissues. Thus, we selected these five miRNAs for further confirmation (Table 1) and found that expression of hsa-miR-337-3p and miR-508-5p was four times greater in the primary cancer tissues compared to the metastatic gastric cancer tissues, while miR-483-5p expression was 2.6 times greater, miR-30c expression was 2.14 times greater, and miR-134 expression was 4.9 times greater in the primary cancer tissues compared to the metastatic gastric cancer tissues (Table 1).

### Loss of hsa-miR-337-3p and hsa-miR-134 expression in metastatic lymph node tumors

**Figure 1:**
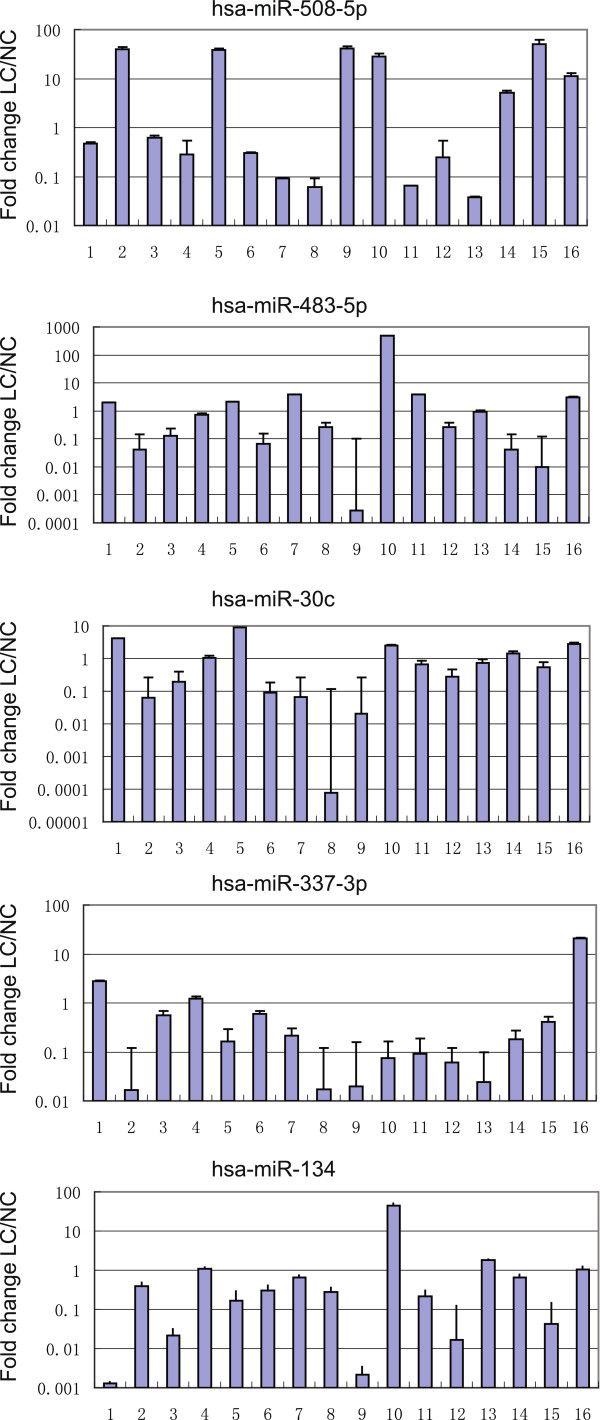
**hsa-miR-508-5p, hsa-miR-483-5p, hsa-miR-134, hsa-miR-30c, and hsa-miR-337-3p in primary gastric cancer and the corresponding metastatic lymph node tissue.** Differential expression of hsa-miR-508-5p **(A)**, hsa-miR-483-5p **(B)**, hsa-miR-134 **(C)**, hsa-miR-30c **(D)**, and hsa-miR-337-3p **(E)** in 16 paired samples of primary gastric cancer (GC) and the corresponding metastatic lymph node tissues (LN) as determined by qRT-PCR. The values shows the fold change of the expression level of LN versus GC (n=3).

Next, we verified these five selected miRNAs in 16 pairs of primary and secondary gastric cancer tissues using qRT-PCR. Our data showed differential expression of hsa-miR-508-5p, hsa-miR-483-5p, hsa-miR-134, hsa-miR-30c, and hsa-miR-337-3p in these 16 paired samples (Figure 1), while expression levels of hsa-miR-337-3p and hsa-miR-134 were significantly reduced in the metastatic tissues compared to the primary gastric cancer tissues (Table 1).

### Expression of hsa-miR-134 and hsa-miR-337-3p in nonmalignant gastric cells and gastric cancer cells

**Figure 2:**
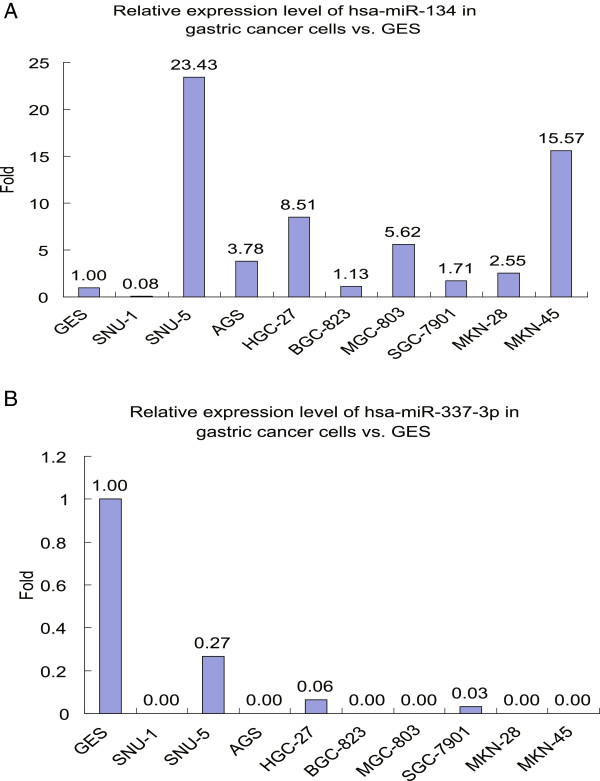
**Expression of hsa-miR-134 and hsa-miR-337-3p in the nonmalignant gastric cell line GES and nine gastric cancer cell lines. A**, hsa-miR-134; **B**, hsa-miR-337-3p.

To determine the potential role of hsa-miR-134 and hsa-miR-337-3p in gastric cancer, we assessed their expression in a nonmalignant gastric cell line (GES) and nine gastric cancer cell lines (SNU-1, SNU-5, AGS, HGC-27, BGC-823, MGC-803, SGC-7901, MKN-28, and MKN-45) using qRT-PCR. As shown in Figure 2A, hsa-miR-134 was highly expressed in SNU-5, AGS, HGC-27, MGC-803, and MKN-28 cells but low in SNU-1 cells compared to GES cells. In contrast, expression of hsa-miR-337-3p was only detected in three gastric cancer cell lines, i.e., SNU-5, HGC27, and SGC-7901, at a low level (Figure 2B).

### Effect of mimics and inhibitors of hsa-miR-134 and hsa-miR-337-3p on MKN-45 cell proliferation

**Figure 3:**
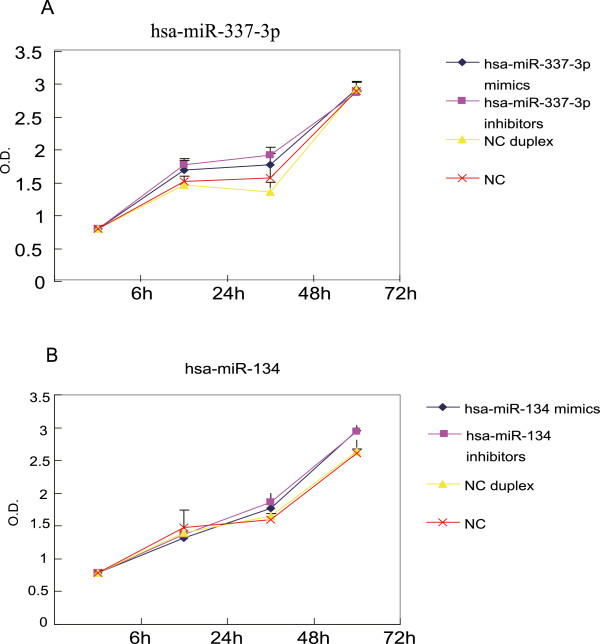
**Time-course effects of miRNAs on the regulation of gastric cancer MKN-45 cell proliferation. A**, hsa-miR-337-3p mimic-transfected MKN-45 cells. **B**, hsa-miR-134 inhibitor-transfected MKN-45 cells. Data are expressed as mean ± SD; n=4.

To determine the effects of hsa-miR-134 and hsa-miR-337-3p on the regulation of gastric cancer growth and invasion, we selected the MKN-45 cell line according to its expression levels of these two miRNAs. The mimic was used to determine whether overexpression of these two miRNAs could inhibit tumor cell invasion *in vitro*, whereas inhibitors were used as controls. (Although they were downregulated in gastric tumor cells, they may have certain levels of expression in tumor cells, and inhibition of their expression may also promote tumor cell invasion.) We transfected hsa-miR-134 or hsa-miR-337-3p mimics or inhibitors into MKN-45 cells and performed a cell viability assay. The data revealed that the changed expression of hsa-miR-134 or hsa-miR-337-3p only slightly affected MKN-45 cell proliferation (Figure 3). miRNA mimics and inhibitors used in this study were listed in Additional file 3: Table S2.

### Expression of hsa-miR-337-3p affects MKN-45 cell migration and invasion

**Figure 4:**
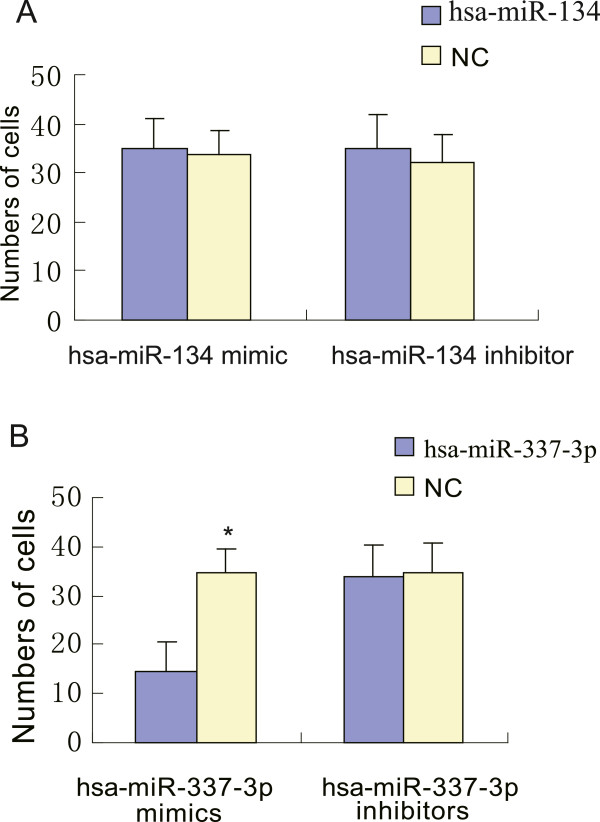
**The effect of hsa-miR-337-3p or hsa-miR-134 mimics or inhibitors on the regulation of gastric cancer cell invasive capacity. A**, The migrated cell number of the hsa-miR-134 inhibitor-transfected MNK-45 cells; **B**: The migrated cell number of the hsa-miR-337-3p mimic-transfected MNK-45 cells. Data are expressed as mean ± SD; n=4; *P<0.05, as compared to the control oligonucleotide (NC) treated group.

Since these miRNAs were differentially expressed in primary and secondary gastric cancer tissues, we investigated the effects of hsa-miR-134 and hsa-miR-337-3p on the regulation of gastric cancer cell migration by transfecting hsa-miR-134 and hsa-miR-337-3p mimics or inhibitors into MKN-45 cells and then measured the tumor cell migration capacity. Next, the capacity of the transfected cells was examined using a Transwell-Matrigel invasion assay. Our data showed that transfection with the hsa-miR-134 mimic or inhibitor in MKN-45 cells did not affect the tumor cell invasion capacity (Figure 4A; P>0.05). In contrast, the hsa-miR-337-3p mimic significantly decreased the number of invaded cells (Figure 4B; P<0.05), indicating that hsa-miR-337-3p overexpression may decrease the invasive ability of gastric cancer cells.

## Discussion

In this study, we profiled the differentially expressed miRNAs between samples of gastric cancer and the corresponding metastatic lymph node gastric cancer tissues using miRNA arrays. We found several miRNAs that were differentially expressed between the two types of samples. Among them, we chose five of the most altered miRNAs to be verified in paired primary and secondary gastric cancers from 16 patients. Next, hsa-miR-134 and hsa-miR-337-3p were transiently transfected into gastric cancer cell lines, and the data showed that they only slightly affected gastric cancer cell growth. However, hsa-miR-337-3p overexpression reduced the invasive ability of gastric cancer cells *in vitro*. Therefore, further studies of the mechanism of hsa-miR-337-3p in gastric cancer are warranted.

Although there are a number of published studies that have investigated aberrant miRNA expression in cancer development and progression *in vitro* and *in vivo*, little research has focused on the altered expression of miRNAs with cancer metastasis [16]. In the present study, we first profiled the altered expression of miRNAs in metastatic lymph node gastric cancer tissues by comparing them with the corresponding primary tumor tissues. We found that more than 400 miRNAs were differentially expressed between these two types of gastric tissues. To date, there have been several studies that have analyzed miRNA expression for its association with gastric cancer or metastasis [8,14-19], and numerous altered miRNA expressions have been reported [14-19], which was confirmed in our current study. However, there have been no reports describing altered miRNA expression between primary gastric cancer tissue and the corresponding metastatic lymph node gastric cancer tissue. Our data support that altered expression of miRNAs does play a role in tumor metastasis. Further studies of these miRNA-targeted genes may provide insightful information for us to understand the molecular mechanisms of tumor metastasis.

Next, we verified 5 miRNAs from the miRNA profiling data in 16 paired gastric cancer tissue samples and in 9 gastric cancer cell lines and found that these miRNA levels were differentially expressed in the tissues and cell lines. Among these five confirmed differentially expressed miRNAs, only miR-483-5p had been previously reported to be associated with human cancer development. For example, Patterson *et al*. showed that altered expression of miR-483-5p is associated with malignant pheochromocytoma after analyzing miRNA expression in benign and malignant pheochromocytoma tumor samples [18]. Using microarray analysis, qPCR confirmation, and Kaplan-Meier analysis, upregulation of miR-483-5p was found to be significant between adrenocortical carcinomas and adrenocortical adenomas [19]. Although our current data are preliminary, this study provides useful information for future studies of miRNAs for their association with gastric cancer metastasis.

Furthermore, our *in vitro* data showed that overexpression of hsa-miR-337-3p in gastric tumor cells reduced tumor cell invasive capacity but only slightly reduced gastric cancer cell proliferation. However, to date, the underlying mechanism for the association of hsa-miR-337-3p with human gastric cancer metastasis is unknown. The hsa-miR-337-3p (miR-337) gene is localized at chromosome 14q32.2. In this chromosome locus, BCL11B may act as a tumor-suppressor gene in T-cell acute lymphoblastic leukemia [20,21]. However, the relationship between hsa-miR-337-3p and BCL11B and their role in gastric cancer metastasis needs to be further determined. Only a few studies have described the role of hsa-miR-337-3p in human tumorigenesis. For example, a previous study has shown that hsa-miR-337-3p is highly expressed in immortalized fetal lung fibroblast IMR-90 cells and is detectable in immortalized human bronchial epithelial HBEC cells [22]. Another study has demonstrated that hsa-miR-337-3p is a modulator of cellular response to taxanes [22]. Furthermore, hsa-miR-337-3p was able to regulate the expression of STAT3 and RAP1A to mediate paclitaxel sensitivity [22]. Indeed, constitutive STAT3 activation is associated with various human cancers and commonly suggests poor prognosis [23,24]. Previous studies have shown that RAP1A is an important player in adhesion and migration of lymphocytes. Moreover, Rap GTPases are master regulators of integrin activation, cell motility, and the underlying cytoskeletal, adhesion, and membrane dynamics. Rap activation is critical for B-lymphoma cells to undergo transendothelial migration *in vitro* and *in vivo*[25]. In addition, altered expression of hsa-miR-337-3p may be critical in renal cell carcinoma (RCC) development, although the analysis of circulating serum levels of hsa-miR-337-3p is unlikely to provide helpful diagnostic/prognostic information in RCC [26]. However, a previous study has reported that hsa-miR-337-3p is among 24 miRNAs that are significantly upregulated in gastric cancer compared to normal gastric mucosae [27], but that study did not specify how many cases were used in the miRNA array analysis and did not verify their results by qRT-PCR [16]. Thus, besides the technological reasons, the previous contradiction of hsa-miR-337-3p expression in gastric cancer can be explained by their different metastatic potentials accordingly to our current findings. Our current study demonstrated that hsa-miR-337-3p acted as a potential therapeutic agent for gastric cancer. For example, we may use a modified hsa-miR-337-3p oligonucleotide mimic to function as hsa-miR-337-3p to inhibit gastric cancer progression and metastasis.

## Conclusions

Our current study demonstrated hsa-miR-337-3p downregulation in metastatic gastric cancer tissues and gastric cancer cell lines. Our *in vitro* study showed that restored hsa-miR-337-3p expression suppressed gastric cancer cell invasion, suggesting that hsa-miR-337-3p may be a potential therapeutic agent to inhibit gastric cancer metastasis.

## Abbreviations

CCK-8: Cell counting kit-8; BSA: Bovine serum albumin; PBS: Phosphate-buffered saline; FBS: Fetal bovine serum.

## Competing interests

The authors confirm that there are no conflicts of interest.

## Authors’ contributions

Conceived and designed the experiments: ZW, JW, and QW. Performed the experiments: ZW and JW. Contributed reagents/materials/analysis tools and analyzed the data: ZW, JW, QW, YY, BH, RW, and YL. Wrote the paper: All authors read and approved the final manuscript.

## Supplementary Material

Additional file 1: Figure S1 Pathology of samples of primary gastric cancer and the corresponding metastatic lymph node tissues. Click here for file

Additional file 2: Table S1 Differential expression of miRNAs between primary gastric cancer and the corresponding metastatic tissue as determined by miRNA expression profile analysis. Click here for file

Additional file 3: Table S2 miRNA mimics and inhibitors used in this study. Click here for file
