# Correction: loss of hsa-miR-337-3p expression is associated with lymph node metastasis of human gastric cancer

**DOI:** 10.1186/1756-9966-33-18

**Published:** 2014-04-11

**Authors:** Zishu Wang, Jin Wang, Yan Yang, Bo Hao, Rui Wang, Yumei Li, Qiong Wu

**Affiliations:** 1 Department of Medical Oncology, The First Affiliated Hospital of Bengbu Medical College, 287 Changhuai Road, Bengbu 233004, Anhui, China; 2 Department of Oncology, Changzheng Hospital, The Second Military Medical University, Shanghai 200433, China; 3 Department of Gastrointestinal Surgery, The First Affiliated Hospital of Bengbu Medical College, Bengbu, Anhui 233004, China

## Correction

After the publication of this work [1], we noticed that we had made a spelling error in title. The word “has-miR-337-3p” in the title should be changed to “hsa-miR-337-3p”. The correct title should be “**Loss of hsa-miR-337-3p expression is associated with lymph node metastasis of human gastric cancer**”.

We sincerely apologized to the readers.
