# Supplementary material for: Loss of has-miR-337-3p expression is associated with lymph node metastasis of human gastric cancer
Source: J Exp Clin Cancer Res. 2013 Oct 16;32(1):76. doi: 10.1186/1756-9966-32-76 (PMC3854519; doi:10.1186/1756-9966-32-76)

poorly diff. adenocarcinoma in stomach

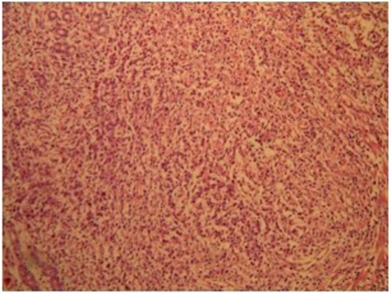

Undifferentiated carcinoma in the lymph nodes

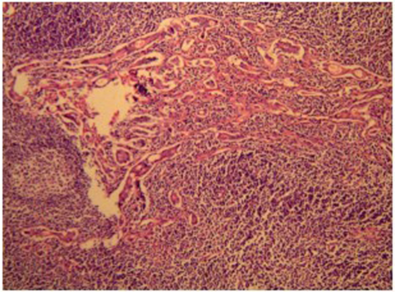

Well diff. adenocarcinoma in the gastric fundus

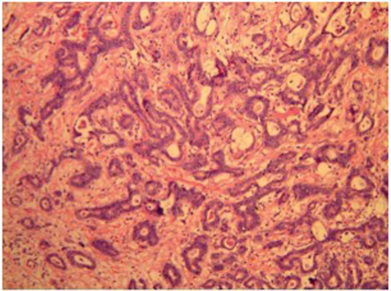

Well diff. carcinoma in ymph nodes

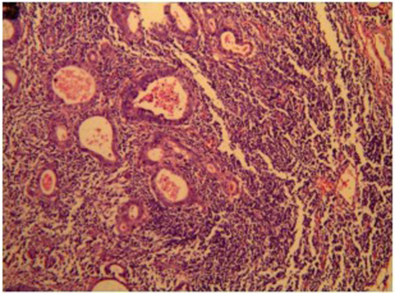

Supplement: Additional file 1: Figure S1 — Pathology of samples of primary gastric cancer and the corresponding metastatic lymph node tissues. [file 1756-9966-32-76-S1.pdf]
