# Supplementary material for: Loss of has-miR-337-3p expression is associated with lymph node metastasis of human gastric cancer
Source: J Exp Clin Cancer Res. 2013 Oct 16;32(1):76. doi: 10.1186/1756-9966-32-76 (PMC3854519; doi:10.1186/1756-9966-32-76)
Supplement: Additional file 2: Table S1 — Differential expression of miRNAs between primary gastric cancer and the corresponding metastatic tissue as determined by miRNA expression profile analysis. [file 1756-9966-32-76-S2.doc]

**Additional file 2: Table S1.** Differential expression of miRNAs between primary gastric cancer and the corresponding metastatic tissue as determined by miRNA expression profile analysis.

| No. | Downregulated miRNA in LN vs. GC  (Fold change ≤0.67 and >0) | Upregulated miRNA in LN vs. GC (Fold change >1.5) |
| --- | --- | --- |
| **1** | hsa-miR-183; hsa-miR-550*; hsa-miR-432; hsa-miR-520b/hsa-miR-520c-3p; hsa-miR-630; hsa-miR-491-5p; hsa-miR-525-5p; hsa-miR-18a*; hsa-miR-875-3p; hsa-miR-103; hsa-miR-330-3p; hsa-miR-503; hsa-miR-98; hsa-miR-885-5p; hsa-miR-181a-2*; hsa-miR-92b*; hsa-miR-557; hsa-miR-498; hsa-miR-342-5p; hsa-miR-337-3p; hsa-miR-34b; hsa-let-7f-1*; hsa-miR-508-5p; hsa-miR-576-3p; hsa-miR-513a-5p; hsa-miR-642; hsa-miR-34c-3p; hsa-miR-302d*; hsa-miR-330-5p;hsa-miR-509-3-5p; hsa-miR-523; hsa-miR-744*; miRPlus_28431; hsa-miR-412; hsa-miR-25*; miRPlus_42530; miRPlus_11239; hsa-miR-383; hsa-miR-20b*; hsa-miR-208a; hsa-miR-425*; hsa-miR-92b; hsa-miR-521; hsa-miR-601; hsa-miR-766; hsa-miR-329; hsa-miR-502-5p; hsa-miR-338-5p; hsa-miR-194*; hsa-miR-892a; hsa-miR-133b; hsa-miR-196a*; hsa-miR-453; hsa-miR-936; hsa-miR-589; hsa-miR-876-3p; hsa-miR-18a; hsa-miR-885-3p; hsa-miR-518f*; hsa-miR-532-3p; hsa-miR-938; hsa-miR-888*; hsa-miR-30c; hsa-miR-345; hsa-miR-134; hsa-miR-185; hsa-miR-519c-5p/hsa-miR-519b-5p/hsa-miR-523*/hsa-miR-518e*/hsa-miR-522*/hsa-miR-519a*; hsa-miR-657; hsa-miR-325; hsa-miR-518a-5p/hsa-miR-527; hsa-miR-487a; hsa-miR-595; hsa-miR-490-5p; hsa-miR-186*; hsa-miR-220c; hsa-miR-640; hsa-miR-150; hsa-miR-520d-5p; hsa-miR-1; hsa-miR-298; hsa-miR-328; hsa-miR-218-2*; hsa-miR-187*; hsa-miR-376a*; hsa-miR-509-5p; hsa-miR-122; hsa-miR-184; hsa-miR-422a; hsa-miR-552; hsa-miR-132*; hsa-miR-99b*; hsa-miR-222*; hsa-miR-877; hsa-miR-548b-3p; hsa-miR-545; hsa-miR-661; hsa-miR-541*; hsa-miR-675; hsa-miR-874; hsa-miR-361-3p; hsa-miR-483-3p; hsa-let-7b*; hsa-miR-138-1*; hsa-miR-138-2*; hsa-miR-27b*; hsa-miR-380*; hsa-miR-512-5p; hsa-miR-130b; hsa-miR-635; hsa-miR-147b; hsa-miR-575; hsa-miR-183*; hsa-miR-147; hsa-miR-617; hsa-miR-934; hsa-miR-615-3p; hsa-miR-299-5p; hsa-miR-378*; hsa-miR-519e*; hsa-miR-610; hsa-miR-500; hsa-miR-300; hsa-miR-623; hsa-miR-485-3p; hsa-miR-197; hsa-miR-124*; hsa-miR-620; hsa-miR-130b*; hsa-miR-135a; hsa-miR-374b*; hsa-miR-483-5p; hsa-miR-490-3p; hsa-miR-29b-2*; hsa-miR-626; hsa-miR-939; hsa-miR-549; hsa-miR-620; hsa-miR-487b; hsa-miR-600; hsa-miR-136; hsa-miR-34c-5p; hsa-miR-515-3p; hsa-miR-381; hsa-miR-627; hsa-miR-99a*; hsa-miR-567; hsa-miR-377; hsa-miR-519d; hsa-miR-518c*; hsa-miR-644; hsa-miR-148a*; hsa-miR-146b-3p; hsa-miR-122*; hsa-miR-338-3p; hsa-miR-326; hsa-miR-340*; hsa-miR-208b; hsa-miR-484; hsa-miR-611; hsa-miR-221*; hsa-miR-424*; hsa-let-7c*; hsa-miR-659; hsa-miR-548b-5p; hsa-miR-125b-2*; hsa-miR-525-3p; hsa-miR-340; hsa-miR-485-5p; hsa-miR-210; hsa-miR-551b; hsa-miR-155*; hsa-miR-628-3p; hsa-miR-7-2*; hsa-miR-206; hsa-miR-507; hsa-miR-367; hsa-miR-337-5p; hsa-miR-516b; hsa-miR-517*; hsa-miR-574-3p; hsa-miR-634; hsa-miR-127-5p; hsa-miR-526b*; hsa-miR-551b*; hsa-miR-553; hsa-miR-190b; hsa-miR-923; hsa-miR-520g; hsa-miR-921; hsa-miR-220b; hsa-miR-584; hsa-miR-30c-2*; hsa-miR-105*; hsa-miR-586; hsa-miR-760; hsa-miR-409-5p; hsa-miR-107; hsa-miR-506; hsa-miR-335*; hsa-miR-361-5p; hsa-miR-603; hsa-miR-125a-3p; hsa-miR-516b*/hsa-miR-516a-3p; hsa-miR-106b*; hsa-miR-612; hsa-miR-551a; hsa-miR-148b*; hsa-miR-450b-3p; hsa-miR-216a; hsa-miR-647; hsa-miR-181d; hsa-miR-671-3p; hsa-miR-519a; hsa-miR-488; hsa-miR-489; hsa-miR-564; hsa-miR-138; hsa-miR-513a-3p; hsa-miR-493 | hsa-miR-126; hsa-miR-199b-5p; hsa-miR-21; hsa-miR-146b-5p; hsa-miR-199a-3p/hsa-miR-199b-3p; hsa-miR-660; hsa-miR-203; hsa-miR-425; hsa-miR-933; hsa-miR-652; hsa-let-7a; hsa-miR-151-3p; hsa-miR-193a-3p; hsa-miR-29c; hsa-miR-195; hsa-miR-30d; hsa-miR-130a; hsa-miR-30a; hsa-miR-143; hsa-miR-200a; hsa-miR-10a; hsa-miR-491-3p; hsa-miR-320; hsa-miR-886-3p; hsa-let-7g; hsa-miR-182; hsa-miR-148b; hsa-miR-191; hsa-miR-29b; hsa-miR-378; hsa-miR-152; hsa-miR-27b; hsa-miR-374a; hsa-miR-126*; hsa-miR-30e; hsa-miR-451; hsa-miR-101; hsa-miR-668; hsa-miR-140-3p; hsa-let-7i; hsa-miR-140-5p; hsa-miR-29a; hsa-miR-421; hsa-miR-886-5p; hsa-miR-27a; hsa-miR-194; hsa-miR-429; hsa-miR-19a; hsa-miR-24; hsa-miR-93; hsa-miR-181a; hsa-miR-125b; hsa-miR-362-5p; hsa-miR-25; hsa-let-7b; hsa-miR-22; hsa-miR-223; hsa-miR-200c; hsa-miR-200b; hsa-miR-16; hsa-miR-30b; hsa-miR-106b; hsa-miR-768-3p; hsa-miR-638; hsa-miR-199a-5p; hsa-miR-20a; hsa-miR-23a; hsa-miR-21*; hsa-miR-636; hsa-miR-26b; hsa-miR-548d-5p; hsa-miR-24-2*; hsa-miR-146a; hsa-miR-222; hsa-miR-151-5p; hsa-miR-155; hsa-miR-100; hsa-miR-145; hsa-miR-28-5p; hsa-miR-129-5p; hsa-miR-106a; hsa-miR-656; hsa-miR-99a; hsa-miR-10b; hsa-miR-142-3p; hsa-miR-221; hsa-miR-186; hsa-miR-744; hsa-miR-26a; hsa-miR-15b; hsa-miR-574-5p; hsa-miR-145*; hsa-miR-342-3p; hsa-miR-768-5p; hsa-miR-214; hsa-miR-30e*; hsa-miR-148a; hsa-miR-23b; hsa-miR-142-5p; hsa-miR-15a; hsa-miR-590-5p; hsa-miR-937; hsa-miR-32*; hsa-miR-801; hsa-miR-17 |
| **2** | hsa-miR-126; hsa-miR-31; hsa-miR-933; hsa-miR-337-3p; hsa-miR-508-5p; hsa-miR-523; hsa-let-7a; hsa-miR-208a; hsa-miR-130a; hsa-miR-143; hsa-miR-766; hsa-miR-200a; hsa-miR-491-3p; hsa-miR-24-1*; hsa-miR-195*; hsa-miR-936; hsa-miR-30c; hsa-miR-345; hsa-miR-134; hsa-miR-378; hsa-miR-30e; hsa-miR-451; hsa-miR-101; hsa-miR-328; hsa-miR-877*; hsa-miR-140-3p; hsa-miR-429; hsa-miR-93; hsa-miR-483-3p; hsa-miR-139-5p; hsa-let-7b; hsa-miR-22; hsa-miR-30d*; hsa-miR-223; hsa-miR-200b; hsa-miR-299-5p; hsa-miR-519e; hsa-miR-106b; hsa-miR-638; hsa-miR-199a-5p; hsa-miR-625*; hsa-miR-23a; hsa-miR-144; hsa-miR-486-5p; hsa-miR-21*; hsa-miR-483-5p; hsa-miR-636; hsa-miR-626; hsa-miR-548d-5p; hsa-miR-24-2*; hsa-miR-16-1*; hsa-miR-627; hsa-miR-146a; hsa-miR-369-3p; hsa-miR-30c-1*; hsa-miR-525-3p; hsa-miR-485-5p; hsa-miR-433; hsa-miR-142-3p; hsa-miR-143*; hsa-miR-520g; hsa-miR-943; hsa-miR-654-5p; hsa-miR-768-5p; hsa-miR-23b; hsa-miR-142-5p; hsa-miR-216a | hsa-miR-886-3p; hsa-miR-193a-5p; hsa-miR-1; hsa-miR-509-5p; hsa-miR-583; hsa-miR-886-5p; hsa-miR-661; hsa-miR-541*; hsa-miR-138-1*; hsa-miR-620; hsa-miR-515-3p; hsa-miR-381; hsa-miR-628-3p; hsa-miR-516b; hsa-miR-214; hsa-miR-361-5p; hsa-miR-647; hsa-miR-181d |
| **3** | hsa-miR-192; hsa-miR-330-3p; hsa-miR-890; hsa-miR-129*; hsa-miR-337-3p; hsa-miR-508-5p; hsa-miR-34c-3p; hsa-miR-143; hsa-miR-329; hsa-miR-886-3p; hsa-miR-597; hsa-miR-30c; hsa-miR-134; hsa-miR-640; hsa-miR-376a*; hsa-miR-886-5p; hsa-miR-635; hsa-miR-548d-3p; hsa-miR-223; hsa-miR-199a-5p; hsa-miR-620; hsa-miR-20a; hsa-miR-23a; hsa-miR-130b*; hsa-miR-135a; hsa-miR-185; hsa-miR-483-5p; hsa-miR-497; hsa-miR-600; hsa-miR-136; hsa-miR-222; hsa-miR-326; hsa-miR-208b; hsa-let-7c*; hsa-miR-125b-2*; hsa-miR-340; hsa-miR-210; hsa-miR-190b; hsa-miR-342-3p; hsa-miR-603; hsa-miR-142-5p; hsa-miR-493 | hsa-miR-193b; hsa-miR-31; hsa-miR-503; hsa-miR-412; hsa-miR-371-5p; hsa-miR-412; hsa-miR-193a-3p; hsa-miR-338-5p; hsa-miR-320; hsa-miR-504; hsa-miR-193a-5p; hsa-miR-186*; hsa-miR-451; hsa-miR-181b; hsa-miR-29a; hsa-miR-602; hsa-miR-125b; hsa-miR-671-5p; hsa-let-7b*; hsa-miR-22; hsa-miR-200c; hsa-miR-16; hsa-miR-30b; hsa-miR-585; hsa-miR-221*; hsa-miR-551b; hsa-miR-645; hsa-miR-23a*; hsa-miR-586; hsa-miR-141; hsa-miR-516b*/hsa-miR-516a-3p; hsa-miR-801 |
