# Supplementary material for: Loss of has-miR-337-3p expression is associated with lymph node metastasis of human gastric cancer
Source: J Exp Clin Cancer Res. 2013 Oct 16;32(1):76. doi: 10.1186/1756-9966-32-76 (PMC3854519; doi:10.1186/1756-9966-32-76)
Supplement: Additional file 3: Table S2 — miRNA mimics and inhibitors used in this study. [file 1756-9966-32-76-S3.doc]

**Additional file 3 Table S2.** miRNA mimics and inhibitors used in this study.

| **miRNA** | **Sequence** |
| --- | --- |
| hsa-miR-134 mimic | Sense 5'-UGUGACUGGUUGACCAGAGGGG-3' |
|  | Antisense 5'-CCUCUGGUCAACCAGUCACAUU-3' |
| hsa-miRNA-134 inhibitor | 5'-CCCCUCUGGUCAACCAGUCACA-3' |
| hsa-miR-337-3p mimic | Sense 5'-CUCCUAUAUGAUGCCUUUCUUC-3' |
|  | Antisense 5'-AGAAAGGCAUCAUAUAGGAGUU-3' |
| hsa-miRNA-337-3p inhibitor | 5'-GAAGAAAGGCAUCAUAUAGGAG-3' |
| NC duplex | Sense 5'-UUCUCCGAACGUGUCACGUTT-3' |
|  | Antisense 5'-ACGUGACACGUUCGGAGAATT-3' |
| NC | 5'-UUGUACUACACAAAAGUACUG-3' |
